# Usual source of care and the quality of primary care: a survey of patients in Guangdong province, China

**DOI:** 10.1186/s12939-015-0189-4

**Published:** 2015-07-31

**Authors:** Zhicheng Du, Yu Liao, Chien-Chou Chen, Yuantao Hao, Ruwei Hu

**Affiliations:** Department of Medical Statistics and Epidemiology, Health Information Research Center, Guangdong Key, Laboratory of Medicine, School of Public Health, Sun Yat-sen University, 74 Zhongshan Road II, Guangzhou, Guangdong Province 510080 Peoples Republic of China; Department of health management, School of Public Health, Sun Yat-sen University, 74 Zhongshan Road II, Guangzhou, Guangdong Province 510080 Peoples Republic of China; Department of International Business, Ling Tung University, Taichung City, Taiwan 40852 R.O.C

**Keywords:** Usual source of care, Primary care, Quality of care, Community health centers

## Abstract

**Introduction:**

Usual source of care (USC) refers to the provider or place a patient consults when sick or in need of medical advice. No studies have been conducted in China to compare the quality of primary care provided with or without USC. The purpose of this study was to fill this gap in the literature by examining the quality of primary care provided between those having a USC and those without. Results of the study would provide implications for policymakers in terms of improving primary care performance in China, and help guide patients in their health care seeking behaviors.

**Methods:**

A cross-sectional survey with patients was conducted in Guangdong province of China, using the Chinese validated Primary Care Assessment Tool (PCAT). ANOVA was performed to compare the overall and ten domains of primary care quality for patients with and without USC. Multivariate analyses were used to assess the association between USC and quality of primary care attributes while controlling for sociodemographic and health care characteristics.

**Results:**

The study added evidence that having a USC can provide higher quality of primary care to patients than those without a USC. Results of this study showed that the PCAT score associated with those having a USC was significantly higher than those not having a USC. Moreover, the study showed that having a usual provider of care was also independently and significantly associated with patients’ satisfaction with care.

**Conclusions:**

This study added evidence that in China, patients with a USC reported higher quality of medical care experiences compared with those without a USC. The efforts to improve quality of care should include policies promoting USC.

## Introduction

Usual source of care (USC) is one of the hallmarks of primary care. It refers to the provider or place a patient consults when sick or in need of medical advice [1]. Internationally, a large body of research indicates that USC could reduce hospitalization cost [2], provide more effective and equitable care [3], increase vaccination coverage [4], enhance timely access to medical care, and improve quality of care received. Studies have also been conducted to examine the effects of USC and demonstrated that USC contributes to better health outcomes [5–7]. The link between USC and quality of care may be explained by the presence of patient-centered care, which is more likely to occur when physicians establish continuing relationships with their patients [8, 9]. The continuing relationship ensures better knowledge of patients as persons rather than symptoms, enhances communication, and reduces chances for conflicts and misunderstandings. Moreover, patient selection of USC has also been associated with higher levels of patient satisfaction with care [10] and a satisfied patient is more likely to follow up with physician orders.

China has a three-tiered health care delivery system, with the primary care system at the bottom, secondary hospitals in the middle, and tertiary hospitals at the top [11, 12]. Despite this classification, patients can access primary care in all facilities across the three levels, having the freedom to choose a doctor or health care facility without the constraints of health insurance policy [13]. Most people prefer higher-level hospitals due to better medical technology and perceived technical quality of the provider, although they will not see the same doctor each time. However, higher-level hospitals require registering in a long queue, which has a significant time constraint. This also means that doctors have less time to treat and interact with their patients. This may result in unsatisfactory experience of care, as patients often complain that their doctors hustle them through appointments.

Studies examining the relationship between USC and quality of care have been conducted primarily in the USA [14–21] and Taiwan [22]. These studies have shown that USC is significantly associated with patients’ experience and perceived quality of care. However, it has been difficult to ascertain whether the observed effects on quality of medical care experiences are in fact due to USC or insurance coverage since many insurance plans require their subscribers to use a USC.

China has been undergoing health care reform, and community health centers (CHCs) are being established as the preferred primary care providers. CHCs make it easier to access a health care provider that may become a USC. Whether this model is able to improve quality of care has come into question. No studies have been conducted in China to compare the quality of primary care provided with or without USC. In Guangdong province, China, a new policy around a family physician model was carried out as a pilot project in that patients are encouraged to sign up with a family physician as their USC. It is necessary to examine the relationship between USC and the quality of primary care in China to provide evidence that USC matters when it comes to delivering quality care and enhance patient satisfaction.

The purpose of this study was to fill this gap in the literature by examining the quality of primary care provided between those having a USC and those without. Results of the study would provide implications for policymakers in terms of improving primary care performance in China, and help guide patients in their health care seeking behaviors and improve provider-patient relationship.

## Methods

### Study setting and design

The study was carried out in Guangdong province of southern China, which is adjacent to Hong Kong and Macau. The population of permanent residents in Guangdong province is more than 100 million, making it the most populous province in China. Variable economic and geographic development makes Guangdong a good case study for China. We conducted a cross-sectional survey with patients who may visit the same doctor/place or shop around different providers when seeking routine medical treatment. The sample size was calculated based on findings from a previous paper that compared the primary care scores between patients at health maintenance organizations and patients at community health centers [23]. The minimum sample size of this study was estimated as 800 with a 95 % confidence interval and a power of 80 %.

A multistage cluster sampling method was adopted. In the first stage, all 21 cities in Guangdong province were categorized into two levels according to the per capita GDP-developed and developing city. In each level, we randomly selected two cities. In each city, we included 200 patients. In the second stage, we stratified between rural and urban areas. In rural areas, we enrolled patients in county hospitals and rural CHCs, while in urban areas we enrolled patients in tertiary hospitals, secondary hospitals and urban CHCs. Thus, there were 50 patients enrolled from each type of health care facility.

Researchers from the School of Public Health of Sun Yat-sen University in Guangdong, China, conducted the primary data collection. Informed consent was obtained from all participating study subjects. The Institutional Review Board (IRB) of Sun Yat-sen University reviewed and approved the protocol of the study in compliance with the Declaration of Helsinki–Ethical Principles for Medical Research Involving Human Subjects (Approval No.: IRB2014.9).

This was a cross-sectional survey using a face-to-face questionnaire administrated by interviewers trained to collect data. The survey was conducted from November 2013 to September 2014. The subject inclusion criteria were as follows: 1) The patient received primary care from the health care facility, 2) The patient usually chose the study site to get primary care if needed, and 3) The patient read the informed consent and agreed to participate in the investigation. The exclusion criteria were as follows: 1) The patient was in poor physical condition and could not complete the survey, or 2) that the patient could not understand the questionnaire.

### Measures

We used the Chinese validated Primary Care Assessment Tool (PCAT) Adult and Child Editions for data collection [24] The PCAT was developed by the Johns Hopkins Primary Care Policy Center to measure the extent and quality of primary care services in provider settings, and is consistent with a focus on attributes of primary care that have been demonstrated to produce better outcomes of care at lower costs [25]. On average, each questionnaire required 20 min to complete. Rather than ratings of satisfaction, PCAT scores objectively measure patients’ experiences with primary care [24].

### Usual source (provider and place) of care

We used two questions in our questionnaire to identify patients with a USC (provider and place): 1) Will you visit the same doctor when you are sick or need advice about your health? 2) Is this facility an appointed medical institution for you?

### Primary care quality

The validated PCAT consists of ten scales representing seven primary care domains: first contact (i.e., access and utilization), longitudinality (i.e., ongoing care), coordination (i.e., referrals and information systems), comprehensiveness (i.e., services available and provided), family centeredness, community orientation, and cultural competence. First contact care implies accessibility to and use of services for each new problem or new episode of a problem for which people seek health care. Longitudinality presupposes the existence of a regular source of care and the characteristics of the interpersonal relationship between that source and the patient. Coordination of care requires some form of continuity, either by practitioners, medical records, or both, as well as recognition of problems that are addressed elsewhere and the integration of their care into the total care of patients. Comprehensiveness implies that primary care facilities must be able to provide or arrange for all types of health care services, including referrals to secondary services for consultation, tertiary services for specific conditions, and essential supporting services such as home care and other community services [26]. Family centeredness, community orientation, and cultural competence refer to the provider’s knowledge of community needs and involvement in the community. These primary care domains are consistent with the US Institute of Medicine’s definition of primary care [27, 28].

For consistency in response and scoring, all items were represented by a Likert-type scale with 1 indicating "Definitely Not," 2 indicating "Probably Not," 3 indicating "Probably," 4 indicating "Definitely," and 9 indicating "Not sure." The sum score for each domain was derived by summing (after reverse-coding where appropriate, 9 was coded with 2.5) the values for all the items under each domain. The sum score for overall quality of primary care experience was derived by summing the values for all domains.

### Satisfaction with care

Satisfaction with care was the outcome in our study. It was coded as a 5-point Likert scale with 1 indicating "very satisfied," 2 indicating "satisfied," 3 indicating "average," 4 indicating "dis-satisfied," and 5 indicating "very dis-satisfied." To simplify the interpretation of results, we collapsed the responses into two categories: satisfied (including 1 and 2) and dis-satisfied (including 3, 4 and 5).

### Sociodemographic and health status

The questionnaire included questions about various sociodemographic characteristics (i.e., gender, age, marriage, rural or urban, household registration, education, occupation, household income) and health status (i.e., self-perceived health status, whether respondent had any physical or mental concerns that lasted for 1 year or longer).

### Analysis

The overall aim of the analysis was to compare the quality of primary care experienced by patients with a USC versus those without a USC. First, we used chi-square to compare sociodemographic and health characteristics of patients with and without a USC. Next, we used paired t-tests to compare quality of primary care indicators for patients with and without a USC. Multiple linear regression models were then used to assess the association between USC and quality of primary care attributes after controlling for patients’ sociodemographic and health characteristics, as well as the type of health care facility. Patient characteristics were included as control variables to account for differences that may lead some patients to choose a USC and others to shop around. Separate models were created for each primary care domain, as well as for overall quality of care. Similarly, two logistic regression models were used to assess the association between satisfaction and USC and overall quality of primary care with and without controlling for the same variables of the multiple linear regression model.

## Results

### Sample characteristics

Table 1 summarizes the general characteristics of the study sample. About half of the respondents were from CHCs (53.13 %). Most respondents were female (59.14 %) and between the ages of 18 and 44 years old (43.52 %). Seventy percent of the respondents were married. More than half of the respondents were from urban area (59 %) and had household registration (53.7 %). About half of the respondents had junior education or lower (47.35 %). Half were unemployed or retired, and 60 % reported an average household income per month, per person between RMB ¥ 1350 and ¥ 4560 (US $220–$745). The majority (76.5 %) of patients reported that they did not have any physical or mental problems, and a significant group (40.16 %) self-assessed their health status as poor.

**Table 1:** Demographic, socioeconomic, and health measures of respondents in Guangdong Province by type of usual source of care

	Total	Usual provider of care = Yes	Usual provider of care = No	Usual place of care = Yes	Usual place of care = No
	(796–864)	(441–471)	(355–393)	(474–500)	(322–364)
	N(%)	N(%)	N(%)	N(%)	N(%)
Facility**, ****					
CHC	459(53.13)	251(53.29)	208(52.93)	303(60.6)	156(42.9)
Secondary hospital	241(27.89)	114(24.2)	127(32.32)	116(23.2)	125(34.3)
Tertiary hospital	164(18.98)	106(22.51)	58(14.76)	81(16.2)	83(22.8)
Gender**, ***					
Female	511(59.14)	300(63.69)	211(53.69)	313(62.6)	198(54.4)
Male	353(40.86)	171(36.31)	182(46.31)	187(37.4)	166(45.6)
Age**, ****					
<18	125(14.47)	66(14.01)	60(15.27)	48(9.6)	78(21.4)
18–44	237(27.43)	184(39.07)	192(48.85)	183(36.6)	193(53)
45–64	376(43.52)	140(29.72)	97(24.68)	165(33)	72(19.8)
≥65	126(14.58)	81(17.2)	44(11.2)	104(20.8)	21(5.8)
Marriage****					
Single	261(30.21)	143(30.36)	118(30.03)	127(25.4)	134(36.8)
Married	603(69.79)	328(69.64)	275(69.97)	373(74.6)	230(63.2)
City class**					
Rural	351(40.63)	155(32.91)	196(49.87)	199(39.8)	152(41.8)
Urban	513(59.38)	316(67.09)	197(50.13)	301(60.2)	212(58.2)
Registered****					
No	400(46.3)	222(47.13)	178(45.29)	167(33.4)	233(64.01)
Yes	464(53.7)	249(52.87)	215(54.71)	333(66.6)	131(35.99)
Education					
Junior or below	402(47.35)	219(47.2)	183(47.53)	244(49.6)	158(44.3)
Senior	168(19.79)	95(20.47)	73(18.96)	88(17.9)	80(22.4)
Technical college	182(21.44)	92(19.83)	90(23.38)	98(19.9)	84(23.5)
Undergraduate or above	97(11.43)	58(12.5)	39(10.13)	62(12.6)	35(9.8)
Occupation*, ***					
Unemployed	438(50.69)	258(54.78)	180(45.8)	255(51)	183(50.3)
Farmer	101(11.69)	46(9.77)	55(13.99)	71(14.2)	30(8.2)
Working in urban	325(37.62)	167(35.46)	158(40.2)	174(34.8)	151(41.5)
Income***					
Low (≤RMB ¥ 1350)	160(20.1)	77(17.46)	83(23.38)	107(22.57)	53(16.46)
Median (RMB ¥ 1350–¥ 4560)	475(59.67)	269(61.00)	206(58.03)	282(59.49)	193(59.94)
High (≥RMB ¥ 4560)	161(20.23)	95(21.54)	66(18.59)	85(17.93)	76(23.60)
Health status*, ****					
Less than good	347(40.16)	206(43.74)	141(35.88)	226(45.2)	121(33.2)
Equal or greater than good	517(59.84)	265(56.26)	252(64.12)	274(54.8)	243(66.8)
Physical/mental problem**, ****					
No	661(76.5)	343(72.82)	318(80.92)	359(71.8)	302(83)
Yes	203(23.5)	128(27.18)	75(19.08)	141(28.2)	62(17)

*P < .05. **P < .01, based on *t* test of difference between usual provider of care

***P < .05. ****P < .01, based on *t* test of difference between usual place of care

Turning to the quality of care indicators shown in Table 2, the highest average score across all patients was in cultural competence (mean = 3.22), followed by utilization (3.05), family centeredness (3.01), coordination of information systems (3), comprehensiveness of services available (2.99), comprehensiveness of services provided (2.86), coordination of referrals (2.77), access (2.75), ongoing care (2.67), and community orientation (2.06).

**Table 2:** Individual and total primary care attributes scores reported by respondents by type of usual source of care

	Total	Usual provider of care = Yes	Usual provider of care = No	Usual place of care = Yes	Usual place of care = No
First contact (utilization)*, ****	3.05 ± 0.64	3.1 ± 0.64	3 ± 0.64	3.14 ± 0.64	2.93 ± 0.63
First contact (access)**, ****	2.75 ± 0.71	2.82 ± 0.68	2.67 ± 0.73	2.86 ± 0.69	2.6 ± 0.71
Ongoing care**, ****	2.67 ± 0.75	2.95 ± 0.66	2.33 ± 0.71	2.78 ± 0.75	2.51 ± 0.72
Coordination (referrals)**, ****	2.77 ± 0.68	2.85 ± 0.69	2.68 ± 0.67	2.83 ± 0.73	2.7 ± 0.61
Coordination (information systems)**, ****	3 ± 0.67	3.12 ± 0.64	2.85 ± 0.69	3.1 ± 0.62	2.86 ± 0.71
Comprehensiveness (services available)****	2.99 ± 0.56	3.02 ± 0.58	2.95 ± 0.54	3.05 ± 0.55	2.89 ± 0.57
Comprehensiveness (services provided)**, ***	2.86 ± 0.76	2.94 ± 0.75	2.76 ± 0.76	2.91 ± 0.78	2.79 ± 0.72
Family centeredness	3.01 ± 0.9	3.05 ± 0.91	2.95 ± 0.9	3.05 ± 0.92	2.94 ± 0.88
Community orientation**, ****	2.06 ± 0.82	2.13 ± 0.84	1.97 ± 0.79	2.15 ± 0.84	1.95 ± 0.78
Cultural competence**	3.22 ± 0.65	3.3 ± 0.64	3.11 ± 0.65	3.24 ± 0.66	3.19 ± 0.64
PCAT total**, ****	28.37 ± 4.52	29.28 ± 4.39	27.28 ± 4.42	29.11 ± 4.57	27.35 ± 4.24

*P < .05. **P < .01, based on *t* test of difference between usual provider of care

***P < .05. ****P < .01, based on *t* test of difference between usual place of cares

### Comparing patients with and without a USC

Comparisons between patients with and without a USC are shown in Table 1. More than half of patients reported having a USC, with 54 % reporting a provider as a USC and 55 % reporting a place as a USC. When considering patients with a provider as a USC, versus patients with no usual provider of care, a greater proportion of patients came from CHCs, were female, between 18 to 44 years of age, reported living in urban areas, were unemployed or retired, did not have poor health status or a physical or mental problem, and reported having a usual provider of care. Similarly, when considering patients with a usual place of care, versus patients with no usual place of care, a greater proportion of patients came from CHCs, were female, between 18 to 44 years of age, married, held household registration, were unemployed or retired, had a median level average household income per month, per person, and did not report poor health status or a physical or mental problem (Table 1).

Looking at quality of care indicators in Table 2, patients with a usual provider of care consistently rated their quality of medical experiences significantly higher than those without a usual provider of care (*P* < 0.05), with the exception of comprehensiveness of services available and family centeredness. When considering a usual place of care, patients rated their quality of medical experiences significantly higher than those without a usual provider of care (*P* < 0.05), except for comprehensiveness of services available.

**Fig. 1:**
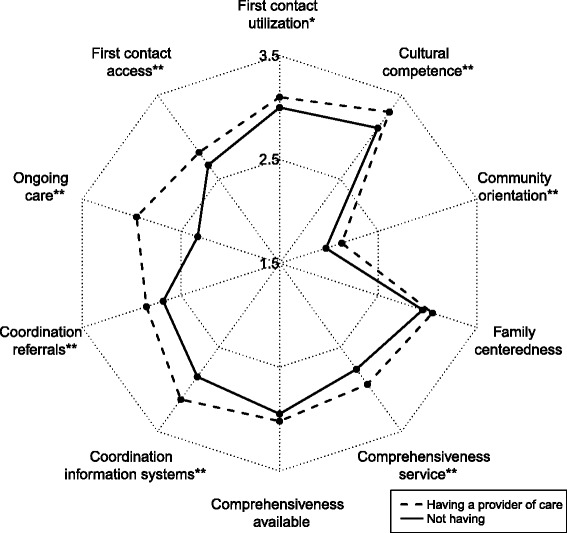
Usual provider of care and primary care attributes. Having a usual provider of care was associated with a higher score on each sub-domain, particularly first contact utilization, first contact access, ongoing care, coordination referrals, coordination information systems, comprehensiveness service, community orientation and cultural competence. Moreover, the scoring gap between having a usual provider of care and not having a usual provider of care in the ongoing care sub-domain was the largest, with scores of 2.95 and 2.33 respectively

**Fig. 2:**
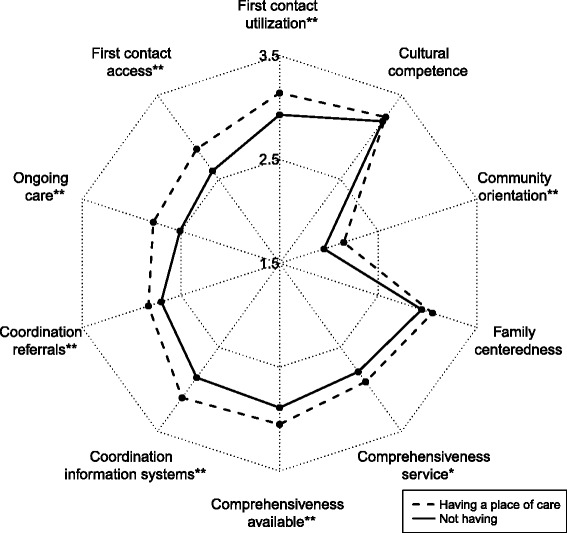
Usual place of care and primary care attributes. Having a usual place of care was associated with a higher score on each sub-domain, particularly first contact utilization, first contact access, ongoing care, coordination referrals, coordination information systems, comprehensiveness available, comprehensiveness service and community orientation. Moreover, the scoring gap between having a usual place of care and not having a usual place of care in the ongoing care sub-domain was the largest, with scores of 2.78 and 2.51 respectively

The radar chart displayed in Fig. 1 shows more detail about the quality of primary care between having and not having a USC. It is apparent that overall, having a usual provider of care was associated with a higher PCAT score than not having one. Figure 1 also provides detailed information on each sub-domain. Having a usual provider of care was associated with a higher score on each sub-domain, particularly first contact utilization, first contact access, ongoing care, coordination referrals, coordination information systems, comprehensiveness service, community orientation and cultural competence. Moreover, Fig. 1 shows that the scoring gap between having a usual provider of care and not having a usual provider of care in the ongoing care sub-domain was the largest, with scores of 2.95 and 2.33 respectively. Figure 2 reveals a similar pattern about usual place of care. Overall, having a usual place of care was associated with a higher PCAT score than not having one. Moreover, patients with a usual place of care scored higher in each sub-domain, with the exception of the family centeredness and cultural competence sub-domains. Likewise, the ongoing care sub-domain had the largest difference (0.27) between those with a usual place of care and those without.

### USC and other predictors of primary care quality

Tables 3 and 4 displays the multiple linear regression coefficients for the association between USC and the ten medical care quality indicators, as well as the PCAT summary score representing overall quality of care, controlling for various socio-demographic, health, and health care characteristics. Patients with a USC reported significantly higher overall quality of medical care experiences than those without a USC (USC Provider: *P* < 0.01, USC Place: *P* < 0.01). Looking at individual quality of care indicators, patients with a usual provider care reported higher quality for all but one indicator (*P* < 0.05). The exception was comprehensiveness of services available, which showed no significant difference between the two groups. Among patients with a usual place care, higher quality was reported for all but two indicators (*P* < 0.05). The exceptions were comprehensiveness of services provided and cultural competence.

**Table 3:** Patient and institutional characteristics associated with individual and total primary care attributes

	First contact utilization	First contact access	Ongoing care	Coordination referrals	Coordination information systems
	Beta(SE)	Beta(SE)	Beta(SE)	Beta(SE)	Beta(SE)
Intercept	3.32**(0.12)	2.75**(0.13)	2.46**(0.12)	2.79**(0.13)	3.01**(0.12)
Usual provider of care (Ref = no)					
Yes	0.1*(0.04)	0.18**(0.05)	0.59**(0.05)	0.2**(0.05)	0.2**(0.04)
Usual place of care (Ref = no)					
Yes	0.18**(0.05)	0.23**(0.05)	0.11*(0.05)	0.11*(0.05)	0.2**(0.05)
Facility (Ref = CHC)					
Secondary hospital	−0.02(0.05)	−0.14**(0.06)	−0.22**(0.06)	−0.11*(0.06)	−0.06(0.05)
Tertiary hospital	−0.01(0.06)	−0.09(0.07)	−0.26**(0.07)	−0.13(0.07)	0.05(0.07)
Gender (Ref = female)					
Male	−0.12**(0.04)	−0.12*(0.05)	−0.03(0.05)	−0.02(0.05)	−0.1*(0.05)
Age (Ref = 65 or older)					
45 to 64	−0.18**(0.07)	0.03(0.08)	−0.03(0.08)	−0.04(0.08)	−0.16*(0.08)
18 to 44	−0.17*(0.08)	0.11(0.09)	−0.07(0.09)	0.005(0.09)	−0.24**(0.08)
Younger than 18	−0.1(0.1)	0.14(0.11)	−0.21*(0.1)	−0.06(0.11)	−0.24*(0.1)
Marriage (Ref = single)					
Married	0.0004(0.06)	0.03(0.07)	−0.12(0.06)	−0.1(0.07)	−0.11(0.06)
City class (Ref = rural)					
Urban	−0.002(0.05)	−0.07(0.06)	0.1(0.06)	−0.04(0.06)	0.13*(0.06)
Registered (Ref = no)					
Yes	−0.02(0.05)	−0.02(0.05)	0.07(0.05)	−0.01(0.05)	−0.04(0.05)
Education (Ref = junior or lower)					
Senior	−0.12*(0.06)	−0.0007(0.07)	−0.09(0.06)	0.02(0.06)	0.02(0.06)
Technical college	0.07(0.06)	0.18**(0.07)	0.13*(0.06)	0.16**(0.07)	0.13*(0.06)
Undergraduate or above	−0.02(0.08)	0.13(0.09)	0.03(0.08)	0.02(0.09)	−0.02(0.08)
Occupation (Ref = unemployed)					
Farmer	0.07(0.08)	0.23**(0.08)	0.03(0.08)	0.16*(0.08)	−0.02(0.08)
Working in urban	−0.06(0.06)	−0.03(0.06)	−0.09(0.06)	−0.03(0.06)	−0.07(0.06)
Income (Ref = low)					
Median	−0.14*(0.06)	−0.17*(0.07)	−0.03(0.07)	−0.1(0.07)	−0.03(0.07)
High	−0.31**(0.08)	−0.34**(0.09)	−0.1(0.09)	−0.25**(0.09)	−0.08(0.08)
Missing	−0.28**(0.09)	−0.31**(0.1)	−0.48**(0.1)	−0.27**(0.1)	−0.28**(0.1)
Health status (Ref = less than good)					
Equal or greater than good	0.04(0.05)	−0.02(0.05)	0.13**(0.05)	0.16**(0.05)	0.15**(0.05)
Health problem (Ref = no)					
Yes	−0.25**(0.06)	−0.2**(0.06)	0.02(0.06)	−0.06(0.06)	−0.04(0.06)
Adjust R squared	0.09	0.10	0.26	0.06	0.11
Sample Size	864	864	864	864	864

*P < .05. **P < .01

**Table 4:** Patient and institutional characteristics associated with individual and total primary care attributes (Cont.)

	Comprehensiveness available	Comprehensiveness service	Family centeredness	Community orientation	Cultural competence	PCAT total
	Beta(SE)	Beta(SE)	Beta(SE)	Beta(SE)	Beta(SE)	Beta(SE)
Intercept	2.94**(0.1)	2.87**(0.14)	2.99**(0.17)	2.22**(0.14)	2.91**(0.12)	28.25**(0.79)
Usual provider of care (Ref = no)						
Yes	0.06(0.04)	0.21**(0.05)	0.15*(0.06)	0.18**(0.05)	0.17**(0.04)	2.03**(0.29)
Usual place of care (Ref = no)						
Yes	0.14**(0.04)	0.08(0.06)	0.13*(0.07)	0.11*(0.06)	−0.03(0.05)	1.26**(0.32)
Facility (Ref = CHC)						
Secondary hospital	−0.25**(0.05)	−0.12(0.06)	−0.07(0.08)	−0.55**(0.06)	−0.15**(0.05)	−1.69**(0.35)
Tertiary hospital	−0.06(0.06)	−0.11(0.08)	0.04(0.09)	−0.53**(0.08)	−0.04(0.07)	−1.14**(0.43)
Gender (Ref = female)						
Male	−0.08*(0.04)	−0.07(0.05)	−0.11(0.06)	−0.03(0.05)	−0.1*(0.05)	−0.79**(0.3)
Age (Ref = 65 or older)						
45 to 64	0.02(0.07)	−0.04(0.09)	−0.09(0.1)	−0.07(0.09)	0.04(0.07)	−0.52(0.49)
18 to 44	0.004(0.07)	−0.05(0.1)	−0.05(0.12)	−0.02(0.1)	−0.03(0.08)	−0.51(0.54)
Younger than 18	0.08(0.09)	−0.18(0.12)	0.18(0.14)	−0.19(0.12)	−0.06(0.1)	−0.64(0.67)
Marriage (Ref = single)						
Married	0.01(0.05)	−0.04(0.07)	−0.01(0.09)	−0.15*(0.07)	0.11(0.06)	−0.39(0.41)
City class (Ref = rural)						
Urban	−0.1*(0.05)	−0.06(0.06)	−0.14(0.08)	−0.03(0.07)	−0.06(0.05)	−0.29(0.36)
Registered (Ref = no)						
Yes	−0.02(0.04)	−0.02(0.06)	−0.06(0.07)	−0.09(0.06)	0.11*(0.05)	−0.11(0.31)
Education (Ref = junior or lower)						
Senior	0.04(0.05)	0.000001(0.07)	−0.05(0.09)	−0.01(0.07)	−0.002(0.06)	−0.19(0.4)
Technical college	0.09(0.05)	0.13(0.07)	0.16(0.09)	0.2**(0.07)	0.08(0.06)	1.33**(0.41)
Undergraduate or above	0.08(0.07)	0.03(0.1)	0.08(0.11)	0.002(0.1)	−0.02(0.08)	0.33(0.54)
Occupation (Ref = unemployed)						
Farmer	0.09(0.07)	0.2*(0.09)	0.34**(0.11)	0.16(0.09)	−0.14(0.08)	1.12*(0.52)
Working in urban	0.03(0.05)	0.02(0.07)	0.1(0.08)	−0.03(0.07)	−0.14*(0.06)	−0.29(0.4)
Income (Ref = low)						
Median	−0.01(0.06)	−0.06(0.08)	−0.08(0.09)	0.02(0.08)	0.16*(0.07)	−0.46(0.43)
High	−0.02(0.07)	−0.2*(0.1)	−0.22(0.12)	−0.24*(0.1)	0.18*(0.08)	−1.6**(0.55)
Missing	0.02(0.08)	−0.14(0.11)	−0.37**(0.13)	−0.17(0.11)	0.02(0.09)	−2.27**(0.63)
Health status (Ref = less than good)						
Equal or greater than good	0.08(0.04)	0.15**(0.06)	0.17**(0.07)	0.25**(0.06)	0.24**(0.05)	1.36**(0.31)
Health problem (Ref = no)						
Yes	0.06(0.05)	−0.12(0.07)	−0.14(0.08)	0.06(0.07)	0.11(0.06)	−0.56(0.38)
Adjust R squared	0.04	0.04	0.05	0.16	0.08	0.16
Sample Size	864	864	864	864	864	864

*P < .05. **P < .01

In addition to USC, other covariates were also significantly associated with the overall quality of medical care, including facility, gender, education, occupation, household income, health status. Specifically, patients at secondary and tertiary hospital reported lower quality of primary care than those at CHCs; male patients reported lower quality than female; those with technical college education reported higher quality than those with junior or lower education; famers reported higher quality than those who were unemployed; those with higher income reported lower quality than those with lower income; those who self-assessed their health status as poor reported lower quality those self-assessed as good.

### Primary care quality, USC, and patient satisfaction

Table 5 displays the logistic regression results for the association between the PCAT score, USC, and patient satisfaction. We conducted two models. Model 1 controlled for USC, while Model 2 controlled for various socio-demographic, health, and health care characteristics as well as USC. Patients with higher total score (*P* < 0.01) and a USC (*P* < 0.05) reported significantly higher satisfaction. In Model 2, even after controlling other predictors, patients with higher total score (*P* < 0.01) and usual provider of care (*P* < 0.05) continued to report significantly higher satisfaction.

**Table 5:** Factors associated with patients’ satisfaction with care

		OR (95 % CI)
Model 1	Intercept	0.02**(0.01,0.07)
	PCAT total	1.19**(1.14,1.25)
	Usual provider of care (Ref = no)	
	Yes	1.52*(1.06,2.18)
	Usual place of care (Ref = no)	
	Yes	1.47*(1.03,2.1)
	Nagelkerke R squared	0.17
	Sample	864
Model 2	Intercept	0.04**(0.01,0.22)
	PCAT total	1.19**(1.14,1.25)
	Usual provider of care (Ref = no)	
	Yes	1.48*(1.01,2.17)
	Usual place of care (Ref = no)	
	Yes	1.02(0.68,1.52)
	Facility (Ref = CHC)	
	Secondary hospital	0.48**(0.32,0.74)
	Tertiary hospital	0.78(0.46,1.34)
	Gender (Ref = female)	
	Male	1.4(0.94,2.09)
	Age (Ref = 65 or older)	
	45 to 64	0.84(0.38,1.79)
	18 to 44	0.57(0.25,1.24)
	Younger than 18	0.24**(0.09,0.6)
	Marriage (Ref = single)	
	Married	0.8(0.45,1.38)
	Registered (Ref = no)	
	Yes	1.55*(1.04,2.31)
	Education (Ref = junior or lower)	
	Senior	0.94(0.57,1.57)
	Technical secondary school or college	1.01(0.6,1.71)
	Undergraduate or above	1.25(0.66,2.44)
	Occupation (Ref = unemployed)	
	Farmer	0.79(0.39,1.64)
	Working in urban	0.92(0.56,1.53)
	Health status (Ref = less than good)	
	Equal or greater than good	1.37(0.91,2.06)
	Health problem (Ref = no)	
	Yes	1.1(0.66,1.87)
	Nagelkerke R squared	0.23
	Sample	864

*P < .05. **P < .01

In Model 2, other covariates were also significantly associated with satisfaction, including facility, age, and household registration. Specifically, patients from secondary hospital reported lower satisfaction than those from CHCs; those younger than 18 reported lower satisfaction than those 65 or older than 65; and those with household registration reported higher than those without.

## Discussion

This study used information collected from patient surveys in Guangdong, China, to explore the effects of USC on the quality of primary care using an internationally and locally validated tool, PCAT. The USC refers to the provider or place a patient consults when sick or in need of medical care. The study added evidence that having a USC can provide higher quality of primary care to patients than those without a USC. Results of this study showed that the PCAT score associated with those having a USC was significantly higher than those not having a USC. Specifically, the PCAT total score for those with a usual provider of care was 29.28, a full two points better than those not having a usual provider of care (27.28). The PCAT total score for those with a usual place of care was 29.11, 1.76 points better than those not having a usual place of care (27.35).

Table 5 shows that, after controlling for other confounders, having a usual provider of care was independently and significantly associated with patients’ satisfaction with care, while having a usual place of care was not statistically significant. This suggests that a usual provider of care may be more important than a usual place of care in influencing patients’ satisfaction with primary care. Patients who were female, 18–44 years old, married, living in urban areas, in a registered home, had a median income and no physical or mental problems were more likely to search for primary care from a usual provider of care and usual place of care.

Although there has been significant research focusing on the association between USC and medical care in other countries [14–22], research on the topic in China is limited. Results of this study demonstrated that after adjusting for confounders, overall quality of primary care was significantly higher for patients with a USC than those without. Our findings are consistent with previous studies that have examined the impact of USC on medical quality [14–22].

Although our study evidence suggested that having a USC can improve quality of primary care, this is not yet a requirement in China and the government imposes no restrictions on health care provider selection. Because of this, health resources may not be effectively used, as patients will crowd in tertiary hospital although their illnesses are not so serious. This may not only reduce the quality of primary care patients receive, but also a waste of health resources. Our study suggested that if there were a health policy guiding patients to use a USC, the overall quality of primary care might improve and the use of health resources could be more appropriate.

Our study also has implications for health care policy in Guangdong, where the proportion of surveyed patients without a USC was 56 %. Based on our study, the government should extent a USC policy, which may contribute to significant increases in quality of primary care. The advantages of USC can not only improve the quality of primary care, but also be conducive to the government in implementing health monitoring and health policy interventions, which may be especially good for the growing number of patients with chronic disease.

It is important to note that although the patients with a USC experienced higher quality of primary care in the community orientation domain, the scores of community orientation were still relatively low even for patients with USC. This suggests that community orientation needs to be improved in the provision of primary care.

There were a number of limitations in this study. First, this study was conducted in one region of China, therefore generalizability of the findings to other regions was restricted. Second, there might be underlying differences between patients who chose a USC and those who chose not to have a USC, which accounted for differences in perceived quality of care. Indeed, our study showed that USC and non-USC patient groups differed regarding age distributions, education levels, and presence of physical or mental problems. Although we controlled for these differences in our regression analyses, there might be other differences that remained unaccounted for. For example, patients who selected CHCs might prefer CHCs while the patients who went to tertiary hospitals might dislike CHCs. Third, this study examined patients’ perceived quality of care experiences, rather than actual outcomes of medical care. Our study was a cross-sectional design, so it may be hard to make causal inferences.

## Conclusions

In conclusion, this study added evidence that in China having a USC can provide higher quality of primary care to patients, which provides basis for health policy that promotes USC. Future studies may explore how to establish and promote the USC policy in China.

## Abbreviations

PCAT: Primary care assessment tool
USC: Usual source of care
